# Co-Inoculation with Arbuscular Mycorrhizal Fungi and Dark Septate Endophytes under Drought Stress: Synergistic or Competitive Effects on Maize Growth, Photosynthesis, Root Hydraulic Properties and Aquaporins?

**DOI:** 10.3390/plants12142596

**Published:** 2023-07-09

**Authors:** Minggui Gong, Na Bai, Pengfei Wang, Jiajie Su, Qingshan Chang, Qiaoming Zhang

**Affiliations:** 1 College of Food and Bioengineering, Henan University of Science and Technology, Luoyang 471023, China; gongminggui@nwsuaf.edu.cn (M.G.); baina817@hotmail.com (N.B.);; 2 College of Horticulture and Plant Protection, Henan University of Science and Technology, Luoyang 471023, China

**Keywords:** fungal endophytes, synergistic effect, drought stress, maize, aquaporins

## Abstract

Arbuscular mycorrhizal fungi (AMF) and dark septate fungi (DSE) were simultaneously colonized in the root cells of maize. Single AMF and DSE symbiosis have been proven to improve the drought tolerance of maize. However, the effects of both fungi coexisting in maize roots under drought stress are not yet known. In this study, pot experiments of maize seedlings were conducted through four inoculation treatments (single AMF inoculation of *Rhizophagus irregularis*, single DSE inoculation of *Exophiala pisciphila*, co-inoculation of AMF + DSE and non-mycorrhizal inoculation) under well-watered (WW) and drought-stressed (DS) conditions. AMF and DSE colonization status, maize physiology and aquaporin gene expression in maize roots were investigated. The objective of this paper was to evaluate whether AMF and DSE had competitive, independent or synergistic effects on regulating the drought tolerance of maize. When maize seedlings of three inoculation treatments were subjected to drought stress, single AMF inoculation had the highest shoot and root dry weight, plant height, root length, osmotic root hydraulic conductivity and hydrostatic root hydraulic conductivity in maize seedlings. However, co-inoculation of AMF + DSE induced the highest stomatal conductance in maize leaves and the lowest H_2_O_2_ and O_2_^•−^ concentration, membrane electrolyte leakage, intercellular CO_2_ concentration and gene expression level of *ZmPIP1;1*, *ZmPIP1;2*, *ZmPIP2;1*, *ZmPIP2;5* and *ZmPIP2;6*. In addition, co-inoculation of AMF + DSE also obviously down-regulated the *GintAQPF1* and *GintAQPF2* expression in *R. irregularis* compared with single AMF inoculation treatment. Under DS stress, there were competitive relationships between AMF and DSE with regard to regulating mycorrhizal colonization, maize growth, root hydraulic conductivity and the gene expression of aquaporins in *R. irregularis*, but there were synergistic relationships with regard to regulating membrane electrolyte leakage, oxidative damage, photosynthesis and the aquaporin gene expression of maize seedlings. The obtained results improve our knowledge about how the mechanisms of AMF and DSE coexist, promoting the drought tolerance of host plants.

## 1. Introduction

Frequent climate change exacerbates drought events in global agricultural production areas [1], and agricultural drought inhibits crop growth and development, reduces crop production and seriously threatens the stability of grain reserves worldwide [2]. To guarantee stable food production, some efforts must be taken to improve the drought resistance of crops, and the underlying drought tolerance mechanisms of crops also need to be profoundly investigated. Drought breaks the balance of crop physiology between root water uptake and leaf transpiration and eventually causes dehydration in crop tissues [3]. Crops often adjust themselves to drought stress through some physiological responses, which include regulating stomatal conductance, photosynthesis, antioxidant production, root hydraulic conductivity and membrane functions [4,5]. Reducing water loss is an essential strategy for crop survival and productivity under drought stress, and cellular water loss in crops also significantly inhibits water permeability by regulating the gene expression of aquaporin related to cellular dehydration tolerance [1,6].

Maize (*Zea mays* L.) is the third most consumed cereal worldwide, after rice and wheat. Its global annual production exceeds one trillion tons, and it is expected to double its demand by 2050 (FAOSTAT) [7]. This C_4_ plant is more susceptible to water deficits and requires more water at the early establishment, vegetative and reproductive stages [8]. Drought stress threatens seed germination and seedling growth, leads to premature flowering and a longer anthesis-silk interval and eventually causes a large-scale reduction in maize production [9]. However, maize has adaptive mechanisms to deal with drought stress, which include enhanced antioxidant activity, presented lower lipid peroxidation, improved osmolyte accumulation, maintained higher photosynthetic activity and root hydraulic conductivity and regulated aquaporin genes [10,11]. 

Arbuscular mycorrhizal fungi (AMF) are a widespread group of root endophytes that establish mutualistic symbiotic relationships with 80% of higher plants [12]. Some literature has shown that AMF protect maize against drought stress by improving water and nutrient absorption, osmotic adjustment, photosynthesis, reactive oxygen species and root formation [13]. Recent evidence has also been found that AMF symbiosis is helpful for the drought tolerance of maize by regulating aquaporin genes in root tissues [7]. Aquaporins, belonging to the plasma membrane intrinsic proteins group (PIPs1 and PIPs2), play a key role in hydraulic permeation and conductivity in plant tissues [14]. The capacity of water transport regulated by aquaporins is about 10–100 times higher than that by free diffusion [15]. The 36 different aquaporin isoforms in maize are found to be ubiquitous membrane channels facilitating the passive transport of water and small soluble substances under low transpiration conditions. Sixteen out of thirty-six maize aquaporins have been proven to be differentially regulated by AMF *Rhizophagus irregularis* according to drought intensity and duration [16]. During drought episodes, water conservation in maize tissues is critical for their growth and productivity, and AMF symbiosis could help drought-sensitive maize to down-regulate some aquaporin genes, which is an effective way to minimize water loss under water deprivation conditions [1]. Therefore, AMF improve the water status and drought tolerance of maize by regulating the gene expression of plant aquaporins [17].

Dark septate fungi (DSE) are characterized by their regular septate, melanized hyphae and melanized microsclerotia [18]. These taxonomically diverse assemblage fungi distribute globally and colonize in diverse plant roots of most terrestrial ecosystems [19]. DSE have been relatively less investigated compared with AMF, and their ecological function for host plants is still under discussion [18]. However, increasing evidence has indicated that DSE symbiosis plays a role in enhancing the drought resistance of maize by promoting its nutrient acquisition, antioxidant enzyme activities, photosynthetic capacity and plant growth [20,21,22]. So far, it is still unknown whether DSE symbiosis regulates the plant aquaporins under normal water and drought conditions.

In the natural ecosystem, root colonization by endophytic fungi is a universal feature in the plant kingdom. AMF and DSE probably constitute the most abundant and widespread group of colonizer endophytes in the rhizosphere [23]. Both fungi often colonize simultaneously in the same root regions and have ecological and functional overlaps [24]. AMF and DSE coexistence in plant roots has been proven to improve soil nutrient cycling and plant development [25]. The growth, P nutrition and antioxidant physiology of maize are found to be improved by AMF and DSE co-inoculation under normal growth conditions [26]. However, the relationships (competition, independence or cooperation) by which AMF and DSE coexist and regulate the growth of host plants have not yet been described. In the present study, AMF and DSE colonization status, maize growth, photosynthesis, oxidative damage and gene expression of *PIP* aquaporin among four inoculation treatments (single AMF inoculation, single DSE inoculation, co-inoculation of AMF + DSE and non-mycorrhizal inoculation) were compared under well-watered (WW) and drought-stressed (DS) conditions. The obtained results improve our knowledge about the mechanisms by which AMF and DSE co-inoculation promotes drought tolerance in maize.

## 2. Results

### 2.1. AMF and DSE Colonization

After inoculation with single AMF, single DSE and dual AMF + DSE, typical AMF (hyphae, spores and arbuscule) or DSE structures were observed, respectively, in the roots of mycorrhizal inoculated maize, but they were not found in the non-inoculated roots (Table 1). The number of AMF spores, the arbuscular colonization rate and the AMF and DSE colonization rates decreased due to drought stress, and these parameters were higher under the WW condition than those under the DS condition. Under both WW and DS conditions, the number of AMF spores and the arbuscule and AMF colonization rates in the single AMF-inoculation treatment were higher than those in the AMF and DSE co-inoculation treatment, and the DSE colonization rate in the single DSE-inoculated roots was also higher than that in the AMF and DSE co-inoculated roots. 

### 2.2. Maize Growth

**Figure 1:**
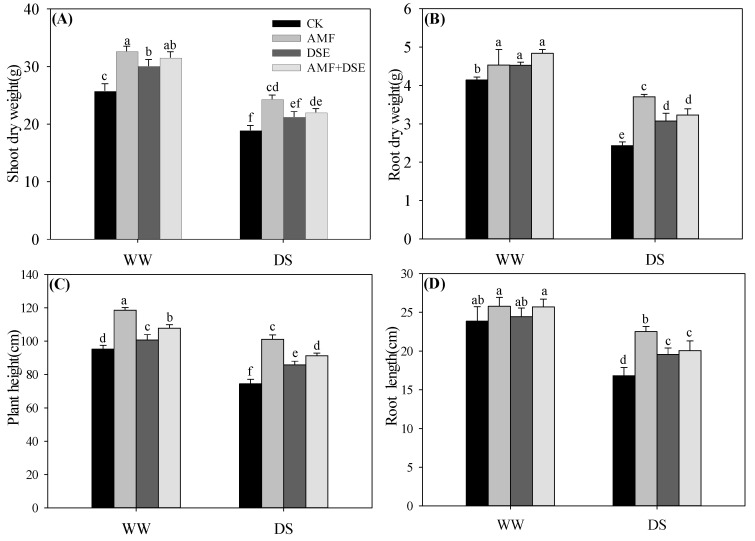
Effects of AMF and DSE on the growth of maize under well-watered (WW) and drought-stressed (DS) conditions. (**A**) Shoot dry weight, (**B**) Root dry dry weight, (**C**) Plant height, (**D**) Root length. CK: non-mycorrhizal inoculation, AMF: *Rhizophagus irregularis* inoculation, DSE: *Exophiala pisciphila* inoculation, AMF + DSE: *R. irregularis* and *E. pisciphila* co-inoculation. The same letter in each column indicates that there was no significant difference among treatments at *p* < 0.05 by using Tukey′s test; values are means ± SD, *n* = 5.

Plant growth was depressed by drought stress, which significantly decreased the shoot and root dry weight, plant height and root length of maize seedlings under the same inoculation condition (Figure 1). Under the WW and DS conditions, the three inoculation treatments had higher maize growth and greater shoot and root dry weight, plant height and root length of maize seedlings than those in the non-inoculation treatment, except that the root length had no apparent difference between non-inoculation and single DSE inoculation treatments under the WW condition (Figure 1D). Under WW and DS conditions, the single AMF inoculation had the highest shoot dry weight and plant height compared to the other two inoculation treatments (Figure 1A,C). Among the three inoculation treatments, the root dry weight was not different under the WW condition, but the root dry weight in the single AMF-inoculated maize was highest under the DS condition (Figure 1B), the root length in the single DSE inoculation treatment was lowest under the WW condition, and in the single AMF inoculation treatment it was highest under the DS condition (Figure 1D).

### 2.3. Membrane Electrolyte Leakage (EL) and Oxidative Damage

**Figure 2:**
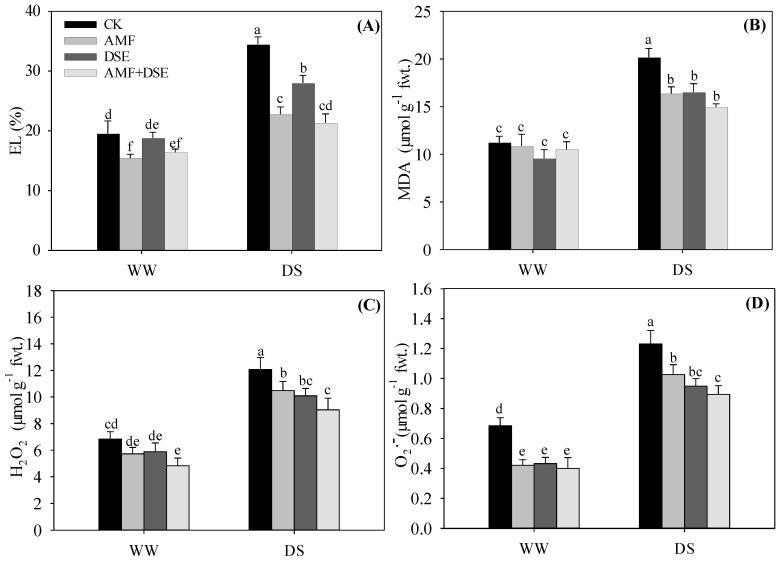
Effects of AMF and DSE on leaf electrolyte leakage and reactive oxygen species of maize under well−watered (WW) and drought–stressed (DS) conditions. CK: non−mycorrhizal inoculation, AMF: *Rhizophagus irregularis* inoculation, DSE: *Exophiala pisciphila* inoculation, AMF + DSE: *R. irregularis* and *E. pisciphila* co–inoculation, (**A**) EL: leaf electrolyte leakage, (**B**) MDA: malondialdehyde, (**C**) H_2_O_2_: hydrogen peroxide, (**D**) O_2_^•−^: superoxide anion. The same letter in each column indicates that there was no significant difference among treatments at *p* < 0.05 by using Tukey′s test; values are means ± SD, *n* = 5.

Drought stress increased the EL under the same inoculation conditions. Under both WW and DS conditions, the EL was reduced by three inoculation treatments compared with non-inoculation treatment (Figure 2A); the EL was highest in the single DSE inoculation treatment and was lowest in the AMF and DSE co-inoculation treatment.

Under the same inoculation condition, drought stress had a negative effect on the MDA, H_2_O_2_ and O_2_^•−^ concentrations in the maize leaves, which were significantly increased by drought stress. The MDA concentration was not different among the four treatments under the WW condition. Under the DS condition, it was highest in the non-inoculated treatment, but had no noticeable difference among the three inoculated treatments (Figure 2B). The H_2_O_2_ concentration in maize leaves was highest in the non-inoculation treatment and was lowest in the AMF + DSE co-inoculation treatment among the four treatments under both WW and DS conditions (Figure 2C). The O2^•−^ concentration had no noticeable difference under the WW condition, but it was the highest in the single AMF inoculation treatment and lowest in the dual AMF + DSE inoculation treatment under the DS condition (Figure 2D) among the three inoculation treatments.

### 2.4. Root Hydraulic Conductivity

**Figure 3:**
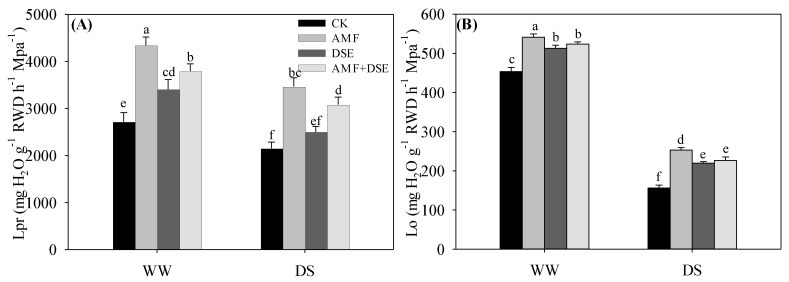
Effects of AMF and DSE on root hydraulic conductivity of maize under well-watered (WW) and drought-stressed (DS) conditions. CK: non-mycorrhizal inoculation, DSE: *Exophiala pisciphila* inoculation, AMF: *Rhizophagus irregularis* inoculation, AMF + DSE: *R. irregularis* and *E. pisciphila* co-inoculation, (**A**) Lpr: Hydrostatic root hydraulic conductivity, (**B**) Lo: Osmotic root hydraulic conductivity. The same letter in each column indicates that there was no significant difference among treatments at *p* < 0.05 by using Tukey′s test; values are means ± SD, *n* = 5.

The Lpr and Lo were significantly reduced by drought stress under the same inoculation treatments (Figure 3). The Lpr and Lo in the non-inoculation treatment were lowest among the four treatments under both WW and DS conditions. The Lpr and Lo were highest in the single-AMF inoculated maize, the Lpr was lowest in the single-DSE inoculated seedlings, and the Lo had no noticeable difference between single DSE and AMF + DSE co-inoculation treatments among the three inoculation treatments under both WW and DS conditions. 

### 2.5. Gas Exchange

**Figure 4:**
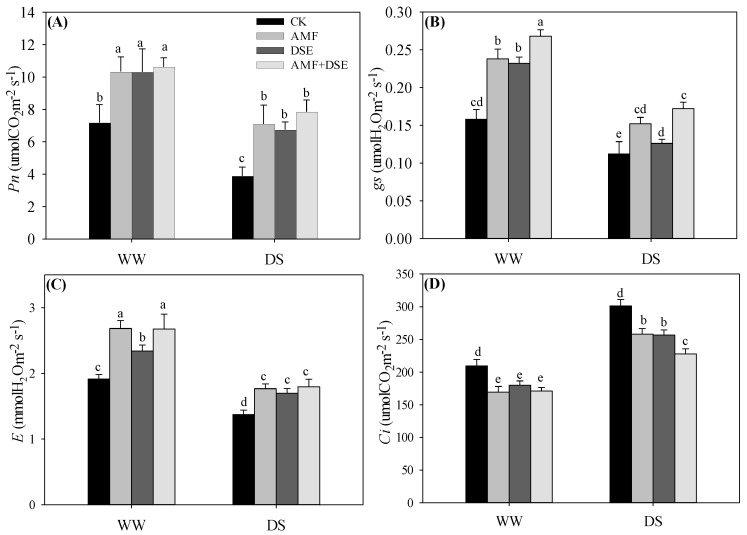
Effects of AMF and DSE on (**A**) net photosynthetic rate (*Pn*), (**B**) stomatal conductance (*g_s_*), (**C**) intercellular CO_2_ concentration (*Ci*) and (**D**) transpiration rate (*E*) in maize leaves under well-watered (WW) and drought-stressed (DS) conditions. CK: non-mycorrhizal inoculation, AMF: *Rhizophagus irregularis* inoculation, DSE: *Exophiala pisciphila* inoculation, AMF + DSE: *R. irregularis* and *E. pisciphila* co-inoculation. The same letter in each column indicates that there was no significant difference among treatments at *p* < 0.05 by using Tukey′s test; values are means ± SD, *n* = 5.

Drought stress had a negative effect on the gas exchange of maize seedlings (Figure 3). It depressed the Pn, gs and E, and increased the Ci under the same inoculation condition. Compared with non-inoculation under both WW and DS conditions, single AMF, single DSE and dual AMF + DSE inoculation remarkably increased the Pn, gs and E and decreased the Ci in maize leaves. The Pn under both WW and DS conditions (Figure 4A), the E under the DS condition (Figure 4C) and the Ci under the WW condition (Figure 4D) were not different among the three inoculated treatments. The gs in AMF + DSE co-inoculated maize was highest among the four treatments under both WW and DS conditions (Figure 4B), and the Ci in AMF + DSE co-inoculated maize was lowest among the three inoculated treatments under the DS condition. The Ci of maize leaves was lowest in the single DSE-inoculated treatment, but it was not remarkably different between single AMF and dual AMF + DSE inoculation among the three inoculated treatments under the WW condition.

### 2.6. Gene Expression of Maize Aquaporins

**Figure 5:**
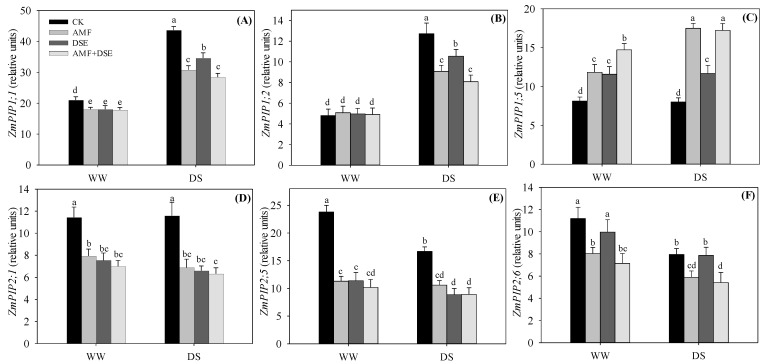
Effects of AMF and DSE on relative expression of PIP aquaporin genes in maize roots under well-watered (WW) and drought-stressed (DS) conditions. (**A**) *ZmPIP1;1*, (**B**) *ZmPIP1;2*, (**C**) *ZmPIP1;5*, (**D**) *ZmPIP2;1*, (**E**) *ZmPIP2;5*, (**F**) *ZmPIP2;6*. CK: non-mycorrhizal inoculation, AMF: *Rhizophagus irregularis* inoculation, DSE: *Exophiala pisciphila* inoculation, AMF + DSE: *R. irregularis* and *E. pisciphila* co-inoculation. The same letter in each column indicates that there was no significant difference among treatments at *p* < 0.05 by using Tukey′s test; values are means ± SD, *n* = 3.

Six *ZmPIP* genes (*ZmPIP1;1, ZmPIP1;2, ZmPIP1;5*, *ZmPIP2;1*, *ZmPIP2;5* and *ZmPIP2;6*) in maize roots that previously were shown to be highly expressed by AMF inoculation were analyzed by qRT-PCR [27]. The expression level of the *ZmPIP1;1* gene was increased by drought stress under the same inoculation conditions, and in the non-inoculated treatment it was also higher than those in the three inoculated treatments (Figure 5A). The expression level of *ZmPIP1;1* was not distinctly different among the three inoculation treatments under the WW condition, but in the single DSE-inoculated treatment it was higher than those in both the single AMF- and dual AMF + DSE co-inoculated treatments under the DS condition.

The *ZmPIP1;2* expression showed a significant increase due to drought stress under the same inoculation condition (Figure 5B). There was no difference in the *ZmPIP1;2* expression among the four treatments under the WW condition. However, under the DS condition, the *ZmPIP1;2* expression was the highest in the non-inoculation treatment and was the lowest in the AMF + DSE co-inoculated treatments among the four treatments.

The *ZmPIP1;5* expression of the non-inoculated and single DSE-inoculated treatments was unaltered, but it increased in both single AMF-inoculated and AMF + DSE co-inoculated treatments by drought stress (Figure 5C). Among the three inoculated treatments, the *ZmPIP1;5* expression was highest in AMF + DSE co-inoculated maize under the WW condition, and it was lowest in single DSE-inoculated maize under the DS condition.

The *ZmPIP2;1* expression in maize roots was not different between the WW and DS conditions, but it showed a significant decrease due to the three inoculation treatments (Figure 5D). Among the three inoculated treatments, the *ZmPIP2;1* expression was highest in the single AMF-inoculated maize and was lowest in AMF + DSE co-inoculated maize under WW and DS conditions.

The *ZmPIP2;5* expression in maize roots was reduced by drought stress under the same inoculation condition and was also decreased due to the three inoculation treatments under both WW and DS conditions (Figure 5E). Among the three inoculated treatments, the *ZmPIP2;5* expression was lowest in the dual AMF + DSE co-inoculated maize under the WW condition, and it was highest in the single AMF-inoculated maize under the DS condition.

Compared with the non-inoculated treatment, the *ZmPIP2;6* expression was not unaltered by single DSE-inoculation, but it was significantly decreased by single AMF- inoculation and dual AMF + DSE co-inoculation (Figure 5F). Among the three inoculated treatments, the *ZmPIP2;6* expression was highest in single DSE-inoculated maize and lowest in dual AMF + DSE co-inoculated maize under both WW and DS conditions.

### 2.7. Gene Expression of AMF Aquaporins

**Figure 6:**
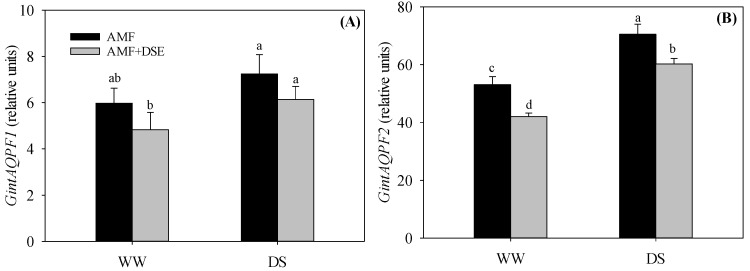
Effects of AMF and DSE on relative expression of (**A**) *GintAQPF1* and (**B**) *GintAQPF2* genes in *R. irregularis* under well-watered (WW) and drought-stressed (DS) conditions. AMF: *Rhizophagus irregularis* inoculation, AMF + DSE: *R. irregularis* and *E. pisciphila* co-inoculation. The same letter in each column indicates that there was no significant difference among treatments at *p* < 0.05 by using Tukey′s test; values are means ± SD, *n* = 3.

The expression of two aquaporin genes (*GintAQPF1* and *GintAQPF2*) in AMF *R. irregularis* from maize roots was analyzed (Figure 6). *GintAQPF1* and *GintAQPF2* were found to be regulated by drought stress and inoculation treatments (*p* < 0.05). *GintAQPF1* and *GintAQPF2* were up-regulated by drought stress under the same inoculation conditions. Compared with single AMF inoculation under both WW and DS conditions, dual AMF + DSE inoculation down-regulated the expression of *GintAQPF1* and *GintAQPF2*. 

## 3. Discussion

Single AMF or DSE inoculation effects on the growth of host plants have been extensively reported in previous studies. Still, there was a lack of knowledge on AMF and DSE co-inoculation under drought stress. Both fungi mutually limited the growth space of mycelium. As a result, the AMF or DSE colonization rate in the co-inoculation treatment was decreased compared with the single AMF or DSE inoculation treatment. The AMF colonization rate was significantly negatively correlated with the DSE colonization rate in *Polylepis australis* roots [28]. AMF symbiosis significantly decreased the DSE colonization rate in ryegrass roots, but DSE did not have a noticeable effect on the AMF colonization rate [29]. The extramatrical phase and presymbiotic stage of AMF (*Gigaspora rosea*) were affected in different ways by DSE (*Dreschlera* sp.), and the exudates from *Dreschlera* sp. stimulated the mycelium extension and branching of *G. rosea* at the presymbiotic stage but inhibited the growth of *G. rosea* at the extramatrical phase [23]. The lower AMF colonization rate at the pre-symbiotic stage was compensated by DSE symbiosis with some weak competitive or antagonistic interaction between both fungal groups [30]. In this study, typical AMF and DSE structures were simultaneously found in AMF and DSE co-colonized maize roots. The number of AMF spores, arbuscules and the AMF colonization rate in the single AMF inoculation treatment and the DSE colonization rate in the single DSE inoculation treatment were higher than those in the AMF and DSE co-inoculation treatments under both WW and DS conditions, which indicated that the two types of fungi might share the ecological niches and compete in the root cells of maize during the symbiotic processes.

Some studies reported that the co-existence of AMF and DSE might establish competitive, independent or collaborative relationships in regulating the growth of host plants [26]. AMF inoculation compensated for the adverse effects of DSE stain *Phialocephala fortinii* on the growth of Norway spruce [31]. Under heavy metal stress, co-inoculation with AMF *Rhizoglomus intraradices* and another endophytic fungus, *Mucor* sp., was more beneficial to the development of wild lettuce than inoculation with a single fungus [32]. In mining waste soils, AMF- and DSE-co-inoculated *Veratrum nigrum* had the maximum growth rate and biomass compared to single AMF- or DSE-inoculated *Verbascum lychnitis* seedlings [33]. Shahabivand et al. also found that dual inoculation with AMF and DSE promoted the growth of wheat in trace element-polluted soil compared with single AMF or DSE inoculation [34]. Under the DS condition in this study, the treatment of single AMF inoculation had the highest shoot dry weight, root dry weight, plant height and root length among the three inoculation treatments, which indicated that AMF and DSE co-inoculation might lead to competitive effects on maize growth, rather than an independent or collaborative relationship under the DS condition. 

The percentage of leaf electrolyte leakage (EL) was often used to evaluate the stability of cell membranes in plants and was a good indicator of plant tolerance to water stress [35]. The EL was significantly increased by 79% in non-inoculated maize, but AMF-inoculated maize maintained a stable state level after drought stress [1]. In this study, the EL% of maize leaves was reduced by the single AMF, single DSE and dual AMF + DSE inoculations compared with the non-inoculation treatment under both WW and DS conditions, which indicated that AMF and DSE symbiosis in maize roots improved the stability of the membranes of maize leave under both well-watered and drought stress conditions. Furthermore, among the three inoculation treatments, the EL% of maize leave was highest in the single DSE inoculation treatment and was lowest in the AMF and DSE co-inoculation treatment, which indicated that AMF and DSE might be competitive rather than independent and collaborative in regulating the electrolyte leakage of maize leaf. The lower EL% showed better membrane stability, which was also related to lower MDA levels [36]. 

The excessive accumulation of MDA in plant cells was often caused by oxidative damage of membrane lipids, and lower MDA levels were usually related to higher membrane stability [9]. The MDA concentration in single AMF-inoculated or AMF + DSE-co-inoculated *Lolium perenne* was significantly lower than that in non-inoculated plants grown in a trace element-polluted soil; in contrast, single DSE inoculation did not alter the MDA concentration of ryegrass [29]. In this study, the MDA concentration in non-inoculated maize was higher than the other three inoculated treatments under the DS condition, but it had no noticeable difference among the four treatments under the WW condition, which showed that AMF and/or DSE inoculation was beneficial for improving the membrane stability of maize cells under DS condition. However, the MDA concentrations in maize leaves were not significantly different among the three inoculated treatments under both WW and DS conditions, which indicated that there were no apparent synergistic effects of both fungal co-inoculations on the MDA concentration in leaves under both WW and DS conditions. 

To cope with water deficits, higher plants regulate the tissues’ permeability to water movement [37]. Osmotic root hydraulic conductivity (Lo) indicates the intrinsic ability of roots to conduct water via the cell-to-cell pathway, and it is highly correlated with the activity and density of water channels between the root surface and xylem in plant stems [1]. Root hydraulic conductivity (Lpr) was used for estimating the root potential of water transport and for determining its role under limited water availability [1]. Khalvati et al. verified that AMF symbiosis regulates the root hydraulic properties in host plants [38]. The increased Lpr and Lo in AMF-inoculated plants under drought stress could be due to the augmentation of the density or size of plasmodesmata by AMF mycelium in plant roots; AMF-inoculated plants do not suffer dehydration as much as non-inoculated plants [39]. In this study, the Lpr and Lo in three inoculation treatments were higher than those in the non-inoculated treatment under both WW and DS conditions, which indicates that DSE symbiosis also regulated the water transport between extracellular and intercellular in roots, providing greater flexibility to host plants for reacting to water deficiency [7]. Among the three inoculation treatments under both WW and DS conditions, the Lpr and Lo were highest in single-AMF inoculated maize, which indicated that there was a competitive relationship between AMF and DSE in regulating the Lpr and Lo of maize.

The increased Lo in AMF-inoculated plants could be related to an increased expression of aquaporins in host plants or AMF [39]. In this study, we also found that the value changes of Lo due to the drought stress and mycorrhizal inoculation treatments did not show complete correlations with the aquaporin gene expression in maize and AMF. Similar results were also reported by Galme et al. and Ruiz-Lozano et al. [6,40]. Some of the reasons may include: (1) The aquaporin activities which directly regulate Lpr and Lo were not only related to the transcriptional level but also to post-transcriptional regulation via phosphorylation, methylation, relocalization or changes in cytosolic pH [6,41]; (2) aquaporins constituted a multiple gene family in plants; only six PIP aquaporin genes were analyzed, and others aquaporins which led to the changes of Lpr and Lo were not analyzed [42]; (3) the Lpr and Lo were not just regulated by aquaporins; they also were significantly affected by the symplastic movement of water via plasmodesmata, depending on the exact environmental circumstances [43].

PIP aquaporins regulated the diffusion of transmembrane water in plant cells through changes in their abundance or channel gating [1]. The effects of AMF symbiosis on the gene expression of aquaporin were complex and more pronounced with intrinsic properties of osmotic stress, plant species and specific aquaporin genes [3]. AMF-regulated aquaporins transported the diversity of substrates and participated in the symbiotic exchange processes between fungi and host plants, which play essential roles in regulating plant physiological processes, such as leaf and root hydraulics, nutrient uptake and transport, stomatal movement, carbon fixation and signaling transduction [7,44]. The mRNA levels of aquaporins genes (*ZmPIP1;1*, *ZmPIP1;3*, *ZmPIP2;2*, *ZmPIP2;4*, *ZmTIP1;1*, *ZmTIP2;3*, *ZmPIP4;1* and *ZmNIP2;1*) in sensitive genotype maize were regulated by AM symbiosis [1]. In this study, we also found that the *ZmPIP1;1*, *ZmPIP2;1*, *ZmPIP2;5* and *ZmPIP2;6* gene expression in maize were also down-regulated by single AMF-inoculation under WW and DS conditions. The down-regulation of aquaporins was a drought tolerance mechanism of plants, which prevented drought damage by decreasing water flow through cell membranes and upholding tissue turgor as a response to water deficit in soils [11]. It is noteworthy that the *ZmPIP1;5* gene expression was even up-regulated by AMF inoculation under WW and DS conditions; this opposite behavior was similar to results reported by Liu et al. or Vinnakota et al. in two rice varieties and two *Malus* species [45,46].

In this study, the *ZmPIP1;1*, *ZmPIP2;1* and *ZmPIP2;5* gene expression in maize were down-regulated by single DSE inoculation under both WW and DS conditions, which indicates that DSE symbiosis had the same effects as AMF, which had a significant effect on the hydraulic conductivity in maize roots through regulating plant aquaporins. We also found that the synergistic effects of AMF and DSE co-inoculation were found in *ZmPIP1;5*, *ZmPIP2;5* and *ZmPIP2;6* gene expression under the WW condition and in *ZmPIP2;1* and *ZmPIP2;6* gene expression under the DS condition. Through inhibiting aquaporin expression, plant tissues kept a tight balance among stomatal movements, root water uptake capacity and water distribution [11,17,47]. Our results indicated that AMF and/or DSE symbiosis could be helping host plants in the above regulation. Moreover, the specific regulation of these aquaporins by AMF and/or DSE symbiosis in maize showed a role in mycorrhiza-induced tolerance to drought stress, which is a possible target for future studies.

AMF regulated the expression of plant aquaporin genes and improved plant water status and drought tolerance [17]. Meanwhile, AMF had their aquaporin genes, and the exploration of the expression characteristics of the aquaporin gene from AMF would be essential to understand the adaptation mechanism of AMF involvement in drought tolerance of host plants. The relative expression of both functional aquaporin genes (*GintAQPF1* and *GintAQPF2*) in *G. intraradices* was identified to be increased under drought stress [48]. Among the three identified aquaporins (*GintAQP1*, *GintAQPF1* and *GintAQPF2*) in *R. irregularis*, GintAQPF2 showed an increased expression under the water deficit condition, which accounted for the improving water transport capacity in mycorrhizal root cells under drought stress [7]. In this study, *GintAQPF1* and *GintAQPF2* were up-regulated by drought stress under the same inoculation conditions, and this result was consistent with Li et al. [48]. The up-regulation of *GintAQPF1* and *GintAQPF2* genes in *G. intraradices* enhanced water transport from the soil to AMF external hyphae [49]. Li et al. also found that both *GintAQPF1* and *GintAQPF2* of AMF *G. intraradices* significantly alleviated the adverse effects of glycerol and polyethylene glycol stress on yeast growth in the heterologous expression experiment [48]. Compared with single AMF inoculation under both WW and DS conditions, AMF and DSE co-inoculation decreased *GintAQPF1* and *GintAQPF2* expression. Both AMF and DSE had some of the same ecological functions in the processes of mutualistic symbiosis with plant roots; therefore, DSE in AMF and DSE co-inoculation treatment undertook the partial task of water transport to plant roots that AMF did in the single AMF-inoculation treatment. Our results showed that AMF and DSE had a competitive relationship during the symbiotic processes in maize roots. In future studies, the response mechanisms of AMF aquaporins to AMF and DSE co-inoculation under drought stress should be addressed.

## 4. Materials and Methods

### 4.1. Experimental Design and Statistical Analysis

A randomized block design was applied in this experiment with two factors: (1) mycorrhizal inoculation treatment, including single inoculation with AMF *Rhizophagus irregularis*, single inoculation with DSE *Exophiala pisciphila*, co-inoculation with AMF and DSE and non-mycorrhizal inoculation; (2) soil water treatment, i.e., well-watered (WW) and drought-stressed (DS) conditions. There were five duplicates of each treatment, giving a total of 40 plants (one seedling per pot).

A two-way ANOVA with inoculation treatment and soil moisture treatment as sources of variation was statistically analyzed by using the SPSS software package (version 13.0 for Windows, SPSS Inc., New York, IL, USA). Tukey’s multiple range test was used to find out whether differences existed among treatment means.

### 4.2. Biological Material

The AMF strain was *Rhizophagus irregularis* (Schenck and Smith), strain BGC BJ09, and it was purchased from the Institute of Plant Nutrition and Resources, Beijing Academy of Agriculture and Forestry Sciences, Beijing, China. The mycorrhizal inoculum comprised sandy soil, spores (spore density was 450 per 10 g dry sandy soil), mycelia and *R. irregularis*-infected maize root segments. Fifty grams of AMF inoculum for the mycorrhizal treatment was positioned 5 cm beneath the maize roots at sowing time. The non-mycorrhizal treatment received 50 grams of sterilized inoculum with a 30 mL filtrate (<20 m) of AMF inoculum for providing a general microbial population free of AMF propagules.

The *Exophiala pisciphila* GM25 strain was supplied by the Key Laboratory of Microbial Resources Exploitation and Utilization, Henan University of Science and Technology, China. It was formerly isolated from the roots of maize (*Zea mays* L.), cultivated onto petri dishes containing potato dextrose agar (PDA) under 28 °C for four weeks and identified according to morphological and molecular biological characteristics. Fresh DSE hyphae in each strain were filtered for inoculum. *E. pisciphila* GM25 inoculum was placed 5 cm below the maize roots. It was covered with gas-permeable plastic film during transplantation, and the non-DSE treatment consisted of sterilized (0.11 Mpa and 121 °C for 2 h) DSE inoculum.

The seeds of a native maize cultivar (*Zea mays* L. cv. genotype Zhengdan 958) were purchased from a seed market in Guanlin Town, Luoyang City, Henan Province. The seeds were surface-sterilized with 0.5% K_2_MnO_4_ for 20 min, washed four times with sterile distilled water under agitation and placed on moist sterilized filter paper in petri dishes at 28 °C and 90% relative humidity for 5 days. After germination to about 1 cm, each germinated seed was transplanted into a plastic container (15 cm in diameter and 15 cm in depth) filled with 3 kg of soil.

### 4.3. Growth Conditions

The soil for culture media in the pot experiment was collected from the topsoil of a botanical garden at Henan University of Science and Technology, China. The physicochemical characteristics of the soil were as follows: pH 8.1 (1:5 soil: water ratio), 0.61% organic matter, 54.61 mg kg^−1^ available potassium, 32.78 mg kg^−1^ available nitrogen and 8.16 mg kg^−1^ Olsen phosphorus. It was sieved (2 mm) and sterilized by steaming (0.11 Mpa and 121 °C for 2 h) before use.

The seedlings were cultivated in a greenhouse between April and July 2019 (temperature range from 20 to 32 °C, 60–90% relative humidity, average photosynthetic photon flux density 900 μmol m^−2^ s^−1^). To avoid inhibiting the symbiosis establishment of AMF and DSE in maize roots, the seedlings were irrigated twice weekly for two months with 100 mL of modified Hoagland nutrient solution (containing only 25% of P) [50]. The DS treatment began two months after the seedling transplantation, and the drought-stressed phase was performed in July 2018 for one month. During this phase, half of the pots were maintained in a well-watered (WW) condition at 70% field capacity (−0.12 MPa), and the other half of the pots were subjected to drought stress (DS) at 35% field capacity (−0.66 MPa) for one month. The water moisture in the soil was maintained by weighing pots and water supplementation every 3 d. The lost water in each pot was supplied daily with fresh distilled water to keep the designed contents of soil water. After one month of the drought-stressed phase, all physiological data were detected.

### 4.4. Measurements

#### 4.4.1. Mycorrhizal Colonization and Plant Growth

To determine the AMF and DSE colonization rates, the maize fine roots were randomly collected, and mycorrhizal fungal colonization was estimated under an optical microscope according to the method of Phillips and Hayman [51]. The gridline intersect method was used to determine the AMF and DSE colonization rates in five replicates for each treatment [52].

At harvest (3 months after sowing), the maize shoots and roots were collected for five replicates per treatment. The plant height and root length were measured by using a steel ruler. The shoots and roots were cut separately, and their dry weight (DW) was measured after drying in a hot-air oven at 70 °C for 48 h.

#### 4.4.2. Membrane Electrolyte Leakage

Leaf electrolyte leakage (EL) was analyzed from five seedlings per treatment. Fresh leaves were cleaned with purified water to remove surface-adhered electrolytes, placed into falcon tubes with 10 mL purified water and incubated on a rotary shaker (at 100 rpm and 25 °C) for 3 h. By using a conductivity meter (Metler Toledo AG 8603, Zurich, Switzerland), the electrical conductivity of the solution (L_0_), final electrical conductivity (L_f_) and conductivity of the deionized water used to incubate the samples (L_water_) were determined according to the method of Quiroga et al. [1]. The EL was defined as follows: EL = [(L_0_ − L_water_)/(L_f_ − L_water_)] × 100.

#### 4.4.3. Root Hydraulic Conductivity

The hydrostatic root hydraulic conductivity (Lpr) was determined at noon with a Scholander pressure chamber 6 h after starting the chemical treatment and following the method described by Bárzana et al. [53]. A gradually increased pressure (0.2, 0.3 and 0.4 MPa) was exerted at 2-min intervals to the detached roots; then sap was collected at the three pressure points. Sap flow was plotted against pressure, and the slope was the root hydraulic conductance (L) value. The Lpr was determined for the root dry weight (RDW) and expressed as mg H_2_O g RDW^−1^ MPa^−1^ h^−1^ [1].

The osmotic root hydraulic conductivity (*L*o) was measured on the detached roots under atmospheric pressure for 2 h by using the free exudation method [54]. The exuded sap was collected and weighed, and a cryoscopic osmometer was used to determine the osmolarity of the exuded sap and the nutrient solution [55]. *L*o was calculated as follows: *L*o = *J*v/ΔΨ, where *J*v was the exuded sap flow rate, and ΔΨ was the osmotic potential difference between the exuded sap and the nutrient solution where the pots were immersed. These measurements were carried out 6 h after the onset of light.

#### 4.4.4. Photosynthetic Efficiency

The efficiency of photosystem II was measured by using the portable photosynthesis system LI-6400 (LI-COR, Lincoln, NE, USA). The net photosynthetic rate (Pn), stomatal conductance (gs), intercellular CO_2_ concentration (*Ci*) and transpiration rate (E) were measured on the second fully expanded leaf of five different plants of each treatment.

#### 4.4.5. Measurement of Oxidative Damage

One gram of fresh leaves or roots was homogenized in 10 mL 10 mM sodium phosphate buffer (pH 7.4) in an ice bath, and the homogenate was subsequently centrifuged at 4000× *g* for 10 min. The malondialdehyde (MDA) concentration was analyzed following the method described by Janero et al. [56]. The rate of H_2_O_2_ and O_2_^•−^ production was determined using the method of Wang and Luo [57]. The absorbance of H_2_O_2_ was spectrophotometrically determined at 390 nm. In order to analyze the O_2_^•−^ concentration, 1 mL of the mixture was added in 1 mL 17 mM sulfanilic acid and 1 mL 7 mM α-naphthylamine for 20 min at 25 °C, and then 3 mL anhydrous was used to leach chlorophyll. Spectrophotometric measurements were performed at 530 nm to determine the concentration of O2^•−^ in the mixture [58].

#### 4.4.6. Gene Expression of PIP Aquaporin

The three *ZmPIP1s* (*ZmPIP1;1*, *ZmPIP1;2* and *ZmPIP1;5*) and three *ZmPIP2s* (*ZmPIP2;1*, *ZmPIP2;5* and *ZmPIP2;6*) genes were chosen for the expression analyses by quantitative real-time PCR because these genes had high-expression levels in maize roots, as found by Hachez et al. [59]. The maize roots were harvested from five independent samples after having been grown for three months and stored at −80 °C. The total RNA of the maize roots was isolated by using a phenol/chloroform extraction and then precipitated with LiCl by using the method of Kay et al. [60]. QuantiTect Reverse Transcription Kit (Qiagen) was used for the RNA reverse transcription according to the instructions, and cDNAs were synthesized by using the method of Ruiz-Lozano et al. [6]. To avoid unspecific gene amplification, the primer sets for the expression analysis of each aquaporin gene were used as reported by Hachez et al. [59]. The expression levels of the maize *α-tubulin* gene were measured for carrying out the standardization of the results [6,59]. The 25 μL reaction system used for the qRT-PCR assay contained 1 μL cDNA (a dilution 1:10), 1 μL each primer, 3 μL SyBR Green, 12.5 μL of SYBR Premix Ex Taq (TaKaRa) and 6.5 μL dH_2_O. RT-PCR was performed by using the CFX96 real-time PCR detection system (Bio-Rad Laboratories, Inc., Hercules, CA, USA). Negative controls without cDNA were used in all PCR reactions. The RT-PCR amplification program of the aquaporin gene and *α-tubulin* gene was initiated at 95 °C for 4 min to activate the polymerase, followed by 35 cycles at 95 °C for 30 s and 60 °C for 60 s for obtaining the target gene, and then the fluorescence signal was measured [6].

The RNA of maize root cells containing arbuscules and germinated spores was extracted by using an RNeasy Plant Mini Kit according to the manufacturer’s instructions, while cDNA was synthesized as described by the method of Li et al. [45]. The fungal aquaporin genes GintAQPF1 and GintAQPF2 were also analyzed using the primers described by Li et al. [45]. The 25 uL reaction medium contained 5 uL of 1:10-diluted cDNA samples and 400 nM gene-specific primers. The PCR program was as follows: one cycle at 95 °C for 30 s and 40 cycles of 5 s at 95 °C, 45 s at 58 °C and 30 s at 72 °C. Standardization was carried out based on the expression of the fungal elongation factor 1a gene in each sample.

The relative abundance of transcripts was calculated by using the 2^−△△Ct^ method [61]. Real-time PCR measurements were carried out in at least five independent RNA samples per treatment, with the threshold cycle (Ct) determined in duplicate. Negative controls without cDNA were used in all PCR reactions.

### 4.5. Statistical Analyses

The statistical analyses were performed by using SPSS statistical software (version 23, New York, IL, USA). The data were analyzed using a two-way analysis of variance (ANOVA). Tukey′s HSD test was used to find out differences among means of treatment at *p* < 0.05.

## 5. Conclusions

In this study, the pot experiments of maize seedlings were conducted with four inoculation treatments under WW and DS conditions. AMF and DSE colonization status, maize physiological parameters and gene expression of aquaporin in maze roots were analyzed. Drought stress suppressed AMF and DSE colonization, maize growth and root hydraulic conductivity and increased membrane electrolyte leakage and oxidative damage in maize leaves, and it regulated the gene expression of PIP aquaporin in maize roots. However, the three inoculation treatments enhanced the drought tolerance of maize by improving the above physiological parameters and down-regulated the gene expression of PIP aquaporin. Under DS stress, AMF and DSE co-inoculation led to competitive effects on mycorrhizal colonization, maize growth and root hydraulic conductivity, but AMF and DSE had a collaborative relationship in decreasing the membrane electrolyte leakage and oxidative damage of maize leaves and improving the photosynthesis of maize and the aquaporin gene expression in maize and AMF. The obtained results improved our knowledge about the mechanisms of AMF and DSE symbiosis in promoting the drought tolerance of host plants. The results may enable future biotechnological applications of AMF and DSE co-inoculation as micro organic complex fertilizers in improving crop growth under drought stress.

## Figures and Tables

**Table 1:** AMF and DSE colonization in maize roots under well-watered (WW) and drought-stressed (DS) conditions.

Water Conditions	Inoculation	Number of AMF Spore (Number /gDW)	Arbuscule Colonization (%)	AMF Colonization(%)	DSE Colonization (%)
WS	CK	-	-	-	-
	AMF	25.67 ± 2.08 a	23 ± 2.00 a	42.42 ± 1.53 a	-
	DSE	-	-	-	42.92 ± 2.57 a
	AMF + DSE	20.67 ± 2.52 ab	17.67 ± 1.53 b	33.42 ± 1.29 b	36.42 ± 1.42 b
DS	CK	-	-	-	-
	AMF	18.33 ± 1.53 b	16.67 ± 1.53 b	29.58 ± 1.88 c	-
	DSE	-	-	-	31.42 ± 1.13 c
	AMF + DSE	12.67 ± 1.53 c	10 ± 1.00 c	18.5 ± 1.00 d	24.33 ± 1.51 d

CK: non-mycorrhizal inoculation, DSE: *Exophiala pisciphila* inoculation, AMF: *Rhizophagus irregularis* inoculation, AMF + DSE: *R. irregularis* and *E. pisciphila* co-inoculation. “-” indicates that the index of the material was not detected. The same letter in each column indicates that there was no significant difference among treatments at *p* < 0.05 by using Tukey′s test; values are means ± SD, *n* = 5.

## Data Availability

Not applicable.
